# Mortality and repellent effects of microbial pathogens on *Coptotermes formosanus* (Isoptera: Rhinotermitidae)

**DOI:** 10.1186/1471-2180-12-291

**Published:** 2012-12-15

**Authors:** Maureen S Wright, Mary L Cornelius

**Affiliations:** 1 United States Department of Agriculture, Southern Regional Research Center, New Orleans, Louisiana, USA; 2 United States Department of Agriculture, Beltsville Agricultural Research Center, Beltsville, Maryland, USA

## Abstract

**Background:**

Two entomopathogenic fungi, *Isaria fumosorosea* and *Metarhizium anisopliae*, and one bacterium, *Bacillus thuringiensis*, were tested for their ability to cause mortality of Formosan subterranean termites (FST), *Coptotermes formosanus* (Shiraki), after liquid exposure, and for their lack of propensity to repel FST.

**Results:**

The fungus *Isaria fumosorosea* at 10^8^ spores/ml caused 72.5% mortality on day 7, significantly higher than the control and 10^6^ spores/ml treatment. On day 14, the 10^6^ and 10^8^ concentrations caused 38.8% and 92.5% mortality, respectively, significantly higher than the control. On day 21, 82.5% and 100% of the termites were killed by the 10^6^ and 10^8^ treatments, respectively. *I. fumosorosea* did not repel termites at 10^6^ nor 10^8^ spores/g in sand, soil or sawdust. The fungus *Metarhizium anisopliae* at 10^8^ spores/ml caused 57.5% mortality on day 7, 77.5% mortality on day 14 and 100% mortality on day 21.

**Conclusions:**

On all three days the rate of mortality was significantly higher than that of the control and 10^6^ spores/ml treatment with *I. fumosorosea*. Neither *I. fumosorosea* nor *M. anisopliae* caused repellency of FST in sand, soil or sawdust. The bacterium *Bacillus thuringiensis* did not cause significant mortality on days 7, 14 or 21. When termites were exposed to cells of *B. thuringiensis* in sawdust and when termites were exposed to a mixture of spores and cells in sand, a significantly higher number remained in the control tubes. Repellency was not seen with *B. thuringiensis* spores alone, nor with the above treatments in the other substrates.

## Background

Microbes have been considered as potential control agents for termites, as alternatives and adjuncts to chemical control measures. Termite behavior and grooming mechanisms present limitations to the effectiveness of termite microbial control [1], though it is suggested that combining pathogenic strains with other strains and with insecticides may improve efficacy [2]. Behavior of mound building termites was found to limit spread of an isolate of *Metarhizium anisopliae* throughout the colony, with repellency being the primary inhibitory factor [3]. A formulation of another strain with reduced repellency was shown to kill nests of *Nasutitermes exitiosus* termites by baiting in limited field trials. The microbes in this study were chosen because of evidence of their causing mortality to termites or other insects and are here screened for their degree of non-repellency.

*M. anisopliae*, when tested against the subterranean termite *Reticulitermes flavipes*, was found to cause alarm, aggregation and defensive reactions among termites that were untreated [4]. Other fungi caused a lesser degree of alarm response which was followed by grooming and isolation of the infected termites. In addition, *M. anisopliae* was found to repel the Formosan subterranean termite (FST), *Coptotermes formosanus*, in tree-based mulches, however some of the repellency may have been attributable to substances from the mulches [5]. Although, potential for *M. anisopliae* as a control agent for termites was demonstrated when, in a test of eight entomopathogenic strains against the subterranean termite *C*. *gestroi*, *M. anisopliae* was found to be the most virulent [6]. A novel strain of *M anisopliae* was found to cause significantly greater mortality of FST alates and workers than a previously commercialized strain [7].

*Isaria fumosorosea* is an entomopathogenic fungus that has been previously shown to cause significant mortality to FST [8]. *I. fumosorosea* is formulated in a wettable powder suitable for delivery with keratin foam. The keratin foam was developed as a biologically compatible delivery mechanism for termite microbial control agents [9,10]. Species of *Paecilomyces* sect. *Isarioidea* are synonymous with *Isaria*[11].

*Bacillus thuringienis* is known to produce compounds toxic to some insects and to be pathogenic to others. Because *Bacillus* strains produce spores there is potential that this microbe will tolerate the nest environment of the termite, and produce infectious propagules in the soil and termite nest environment inhabited by termites. *B. thuringiensis* Berliner has caused mortality of the termite *Nasutitermes ehrhardti*[12]. *Bacillus* isolates have been identified in the gut of *C. formosanus*, indicating the ability of the genus to survive, and potentially cause mortality of the termite [13].

Termite antennae play a significant role in grooming [14]. Termites without antennae did not remove conidia of *I. fumosorosea* and *M. anisopliae* as efficiently as did termites with antennae. Also, termites reared individually were more susceptible to microbial infection than were termites reared in groups and subject to grooming by nestmates [15,16]. To effectively control termites using microbes it will be critical to select pathogens that are capable of not only causing mortality but also withstanding detection and removal. Microbial strains that are both virulent and non-repellent have a greater likelihood of being spread within a termite nest and controlling termites in the field. Results are described here for virulence and non-repellency of potential microbial control strains.

## Results and Discussion

A concern when applying microbial control agents is whether they will repel the target insect rather than infect and kill them. Studies with termites in the laboratory show the ability of microbial agents to kill termites, however few of these experiments have been translated to the field [1,3,17]. FST are known to remove infected nestmates from the nest and to partition infected areas of the nest and this has the potential to limit availability of inoculum [1,15]. By selecting strains of fungi and bacteria that are pathogenic and also not repellent to termites, the probability of applying a microbial agent that functions successfully in the field is increased.

**Figure 1:**
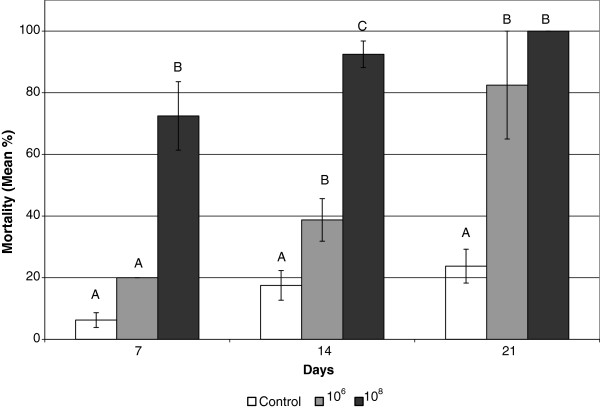
**Mean mortality of Formosan subterranean termites by *Isaria fumosorosea *spore solutions.** Bars on the same day with the same letter are not significantly different.

*I. fumosorosea* is known to cause mortality of insect pests [8,18]. A fermentation method was developed to produce stable spores in an inert powder which can be wetted, thereby inducing germination, prior to application [19]. This powder formulation has been combined with a biologically-compatible foam to permit expansion of the pathogen into the carton nest of termites [9]. Foam expansion increases exposure of termites to the fungal pathogen carried therein. *I. fumosorosea* was previously shown to kill termites which were exposed directly to the dry formulation powder [8]. To more closely approximate field application of a wet microbial agent, termites were exposed to the spores in a liquid solution, as opposed to a dry formulation. The termites were transferred from the liquid to dampened filter paper, which served as a moisture and nutrient source, for incubation and enumeration of mortality. By day 7 the 10^6^ and 10^8^ spores/ml treatments caused 20.0 ± 0% and 72.5 ± 11.1% mortality, respectively (Figure 1). Upon calculating the analysis of variance it was determined that the 10^6^ treatment was not significantly different from the control which caused 6.3 ± 2.4% mortality on day 7. On day 14, the control had reached 17.5 ± 4.8% mortality, while the 10^6^ and 10^8^ concentrations had reached 38.8 ± 6.9% and 92.5 ± 4.3%, respectively. All three mortality rates were significantly different from each other on day 14. On day 21, the 10^6^ and 10^8^ concentrations caused mortality rates of 82.5 ± 17.5% and 100 ± 0%, respectively, which were not significantly different from each other, but they were both significantly different from the control mortality rate of 23.8 ± 5.5%.

**Figure 2:**
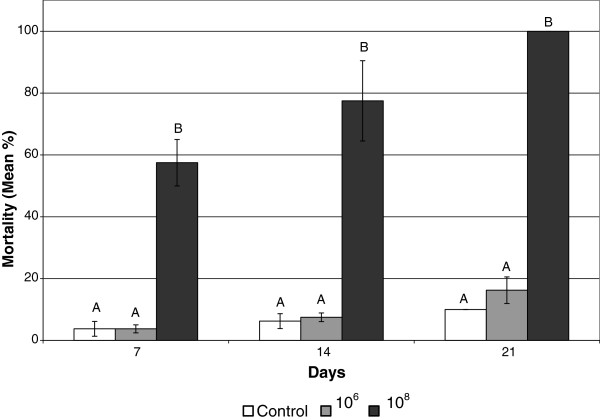
**Mean mortality of Formosan subterranean termites by *Metarhizium anisopliae *spore solutions.** Bars on the same day with the same letter are not significantly different.

*M. anisopliae* strain NRRL 30905 was isolated from dead FST alates and was found to be pathogenic to both FST alates and workers [7]. Spores were previously introduced to termites by individual inoculation [7]. Using the liquid exposure method it was found that on day 7 the 10^8^ spores/ml concentration caused 57.5 ± 7.5% mortality, which was significantly higher than the 3.8 ± 2.4% and 3.8 ± 1.25% mortality exhibited by the control and the 10^6^ spores/ml concentration, respectively (Figure 2). On day 14, the control and 10^6^ spores/ml concentration were again not significantly different at 6.3 ± 2.4% and 7.5 ± 1.4%, respectively, while the 10^8^ spores/ml concentration caused 77.5 ± 13.0% mortality. By day 21 the 10^8^ spores/ml concentration had killed 100 ± 0% of the termites and the 10^6^ spores/ml treatment, at 16.3 ± 4.3% mortality, was still not significantly higher than the control mortality which was 10.0 ± 0% (Figure 2).

**Figure 3:**
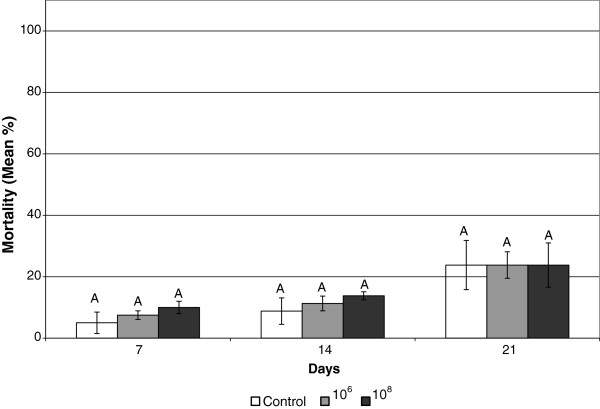
**Mean mortality of Formosan subterranean termites by *Bacillus thuringiensis *spore solutions.** Bars on the same day with the same letter are not significantly different.

*B. thuringiensis* strain 33679 was selected from a culture collection for evaluation against FST. It was originally isolated from diseased insect larvae. Neither of the *Bacillus* treatments caused significantly higher mortality than the control on days 7, 14 or 21 (Figure 3). On day 21 the mortality rate was 23.8 ± 8.0% for the control, 23.8 ± 4.3% for the10^6^ treatment and 23.8 ± 7.2% for the 10^8^ treatment. On day 7 the control caused 5.0 ± 3.5% mortality, 10^6^ cells/ml caused 7.5 ± 1.4% mortality, and 10^8^ cells/ml caused 10 ± 2.0% mortality. On day 14, the mortality values for the control, the 10^6^ and 10^8^ treatments were 8.8 ± 4.3%, 11.3 ± 2.4% and 13.8 ± 1.3%, respectively.

Each of the microbial agents was evaluated for the degree of non-repellency toward termites. Non-repellent agents are less likely to be detected and avoided by termites, thereby increasing the probability of causing a pathogenic effect [20]. Termites were tested by exposure to the three microbes in sand, soil and sawdust. The number of FST remaining in tubes containing an entomopathogen was compared to the number of termites remaining in control tubes following 24 hrs in a paired choice test. Repellency was evident by termite foraging behavior in treated arenas differing significantly from termite behavior in untreated controls. Non-repellency was reported as no statistical difference between the numbers of termites in tubes.

There were no significant differences when termites were exposed to *I. fumosorosea* at either 10^6^ spores/g or 10^8^ spores/g in any of the substrates (Table 1). At a concentration of 10^8^ *M. anisopliae* spores/g, an average of 12.3 ± 2.0 termites remained in the treated sand tubes while 23.0 ± 5.9 remained in the controls, but the difference was not significant. With some treatments, ex. *I. fumosorosea* and *M. anisopliae* in soil and sawdust, more termites remained in treated tubes after 24 h exposure than in control tubes, but none of the treatments was significantly different from its respective control. Based on these data the fungi *I. fumosorosea* and *M. anisopliae* were shown to not be repellent to FST in sand, soil or sawdust.

**Table 1:** **Mean (±SEM) number of *C. formosanus *in a paired choice test where tubes were filled with substrate treated with fungal spores at the indicated concentrations, after 24 h exposure**

	**Number of termite in tubes**	
**Treatment**	**Treated**	**Control**
***I. fumosorosea *10**^**6 **^**spores/g**		
Sand	36.3 ± 13.5a*	60.2 ± 17.3a
Soil	96.1 ± 11.1a	77.4 ± 10.6a
Sawdust	92.5 ± 9.6a	72.8 ± 10.2a
***I. fumosorosea *10**^**8 **^**spores/g**		
Sand	46.0 ± 6.5a	50.8 ± 4.5a
Soil	71.3 ± 16.0a	82.7 ± 17.1a
Sawdust	49.3 ± 9.8a	56.1 ± 9.7a
***M. anisopliae *10**^**6 **^**spores/g**		
Sand	23.9 ± 5.5a	45.0 ± 13.0a
Soil	82.3 ± 7.4a	76.0 ± 7.0a
Sawdust	93.4 ± 9.2a	62.7 ± 9.3a
***M. anisopliae *10**^**8 **^**spores/g**		
Sand	12.3 ± 2.0a	23.0 ± 5.9a
Soil	78.3 ± 12.6a	77.6 ± 12.8a
Sawdust	31.0 ± 3.9a	36.5 ± 4.5a

* Values with the same letter are not significantly different, P ≤ 0.05.

When termites were exposed to *B. thuringiensis* strain 33679 the effect of both cells and spores was determined. All treatments were applied at a concentration of 10^9^ propagules/g. With cells in sand or soil, the treated tube values were not significantly different from the controls (Table 2). With cells in sawdust, the difference was highly significant with only 29.3 ± 6.6 termites remaining in the treated tubes compared with 130.8 ± 9.6 in the control tubes (Paired choice t-test). These values indicated that the *B. thuringiensis* cells were strongly repellent to FST in sawdust. FST were also exposed to a *B. thuringiensis* culture in which the cells had formed spores due to nutrient deprivation. Neither the soil nor sawdust treatments were significantly different from the respective controls, indicating that *B. thuringiensis* in these treatments was not repellent to FST. *B. thuringiensis* was also tested for its effect on FST as a mixture of cells and spores. The culture was incubated in media with a diluted nutrient source and the formation of spores was observed microscopically over time. The termites were exposed when the culture was as close as possible to 50% vegetative cells and 50% spores. In sand, the cell/spore treatment resulted in significantly more termites remaining in the control tubes compared with the treated tubes. Neither the soil or sawdust treatments were significantly different from the controls.

**Table 2:** **Mean (±SEM) number of *C. formosanus *in a paired choice test where tubes were filled with substrate treated with *Bacillus thuringiensis *strain ATCC 33679 at a concentration of 10**^**9 **^**propagules/g, after 24 h exposure**

	**Number of termites in tubes**	
**Treatment**	**Treated**	**Control**
**Cells**		
Sand	43.5 ± 15.0a*	66.5 ± 17.1a
Soil	51.6 ± 8.9a	82.0 ± 10.9a
Sawdust	29.3 ± 6.6a	130.8 ± 9.6b
**Spores**		
Sand	32.9 ±14.3a	26.1 ± 6.7a
Soil	70.2 ± 10.6a	77.1 ± 12.2a
Sawdust	65.8 ± 7.3a	70.5 ± 13.8a
**50**% **Cells + 50**% **Spores**		
Sand	31.5 ± 4.4a	88.3 ± 12.3b
Soil	41.1 ± 8.4a	60.3 ± 12.6a
Sawdust	66.3 ± 11.9a	66.8 ± 12.0a

* Values with the same letter are not significantly different, P ≤ 0.05.

## Conclusions

Of the microbes tested, *I. fumosorosea* demonstrated the highest rate of mortality when termites were exposed to the spores in liquid. This is consistent with previous mortality studies that showed a significant pathogenic effect of this fungus against FST [8,18]. In this study *I. fumosorosea* was also found to not repel termites in a paired choice test in sand, soil or sawdust. For any microbial agent to be effective as a termite control agent the cells or spores must not be repellent, as repellency will result in detection and avoidance by the members of the colony [20]. *I. fumosorosea* has the added advantage of being produced as a stable powder [19]. This fungus has also been formulated in a biologically-compatible foam suitable for application to termite nest environments [9]. The foam has the potential to be used with *M. anisopliae* and other microbial agents.

Of the microbes tested, *B. thuringiensis* cells were found to repel termites only when in sawdust, and in the combination of cells and spores in sand. The remaining treatments, cells in sand and soil; spores in sand, soil and sawdust; and a combination of cells and spores in soil and sawdust, were not repellent to FST. However, when termites were exposed in liquid to the bacterium it was found to not be significantly pathogenic.

Based on the data reported here the fungi tested were found to not be repellent to FST. Both strains are pathogenic to this species of termite and have potential to control it in the field. The *Bacillus* strain had the lowest rate of mortality and, when exposed as cells in sawdust or as a combination of cells and spores in sand, was repellent to FST. Of the three microbes tested it would be the least likely to be selected for further development. The method reported here can be used to screen other *Bacillus* strains, and other potential bacterial entomopathogens, for mortality of FST in liquid. Using this method more closely approximates the liquid-based application which will ultimately be used in the field. The fact that the *I. fumosorosea* and *M. anisopliae* strains tested were pathogenic to FST and were here found to not repel termites makes them viable candidates for control of FST.

## Methods

*Isaria fumosorosea* strain ARSEF 3581 was provided as blastospores in a wettable powder formulation with kaolin clay as the inert carrier by Dr. Mark Jackson (NCAUR, Peoria, IL) [19]. *Metarhizium anisopliae* strain NRRL 30905 (ARS Patent Culture Collection, Peoria, IL, USA) was isolated in this laboratory from dead FST alates [7]. It was inoculated onto potato dextrose agar (PDA) plates and incubated at 25°C for 7 d. Spores were harvested from the plates by scraping with a sterile loop. *Bacillus thuringiensis* Berliner strain ATCC 33679, isolated from diseased insect larvae, was obtained from the American Type Culture Collection (Manassas, VA, USA). A 100 μl aliquot of cells was removed from a tube stored at −80°C and used to inoculate 10 ml of LB. The culture was incubated at 28°C and 225 rpm for approx 6 hr, then used to inoculate 100 ml of LB which was incubated at 28°C and 225 rpm overnight. To encourage spore formation, a 10 ml culture of *B. thuringiensis* in LB was used to inoculate 100 ml of LB prepared at 25% (w/v) of the manufacturer’s standard recipe. The bacterial mass was harvested by centrifugation at 13 krpm for 20 min at 4°C in an angle rotor. The pellet was resuspended in water. Fungal spores, and bacterial cells and spores were enumerated using a Levy hemacytometer (0.1 mm deep; VWR, West Chester, PA, USA). *B. thuringiensis* cultures were determined to have reached 50% cells + 50% spores, and 100% spores by enumeration using the hemacytometer.

Termites were collected from City Park, New Orleans, LA from bucket traps [21]. Four colonies were used for each treatment to prevent colony vitality biasing of data. Twenty FST from each colony were placed into a 2 ml conical microcentrifuge tube containing 0.5 ml of the spore/cell solution for 2 minutes, independent of termites from the other colonies. Tubes were agitated by hand during the incubation time to ensure that the termites were submerged in the liquid. The termites were then transferred to a 90 mm disc of filter paper (Whatman, Maidstone, England) in the lid of a 100 × 15 mm Petri dish where they were allowed to air dry. Control termites were exposed as described above, but the microcentrifuge tube contained water only without the addition of spores or cells. The termites were then transferred to a 55 mm Whatman filter paper disc moistened with water, which served as a moisture and nutrient source, and placed in the lid of a 60 × 15 mm Petri dish. Termites were incubated at 25°C and 85% humidity while mortality was monitored.

Termites were kept in the lab in 5.6-L covered plastic boxes containing moist sand and blocks of spruce *Picea* sp. until they were used in experiments. Treated substrates (sand, soil, or red oak sawdust) were inoculated with the stated concentration of microbe (w/w) and placed in a ½ gallon plastic bottle (Nalgene, Rochester, NY, USA). The bottle was rotated at 2 rpm (80% motor speed) for 6 hrs on a Wheaton Roller Apparatus (Millville, NJ, USA) at room temperature to ensure even distribution of cells and/or spores prior to transfer to the test containers. Control substrates did not contain any of the microbes.

**Figure 4:**
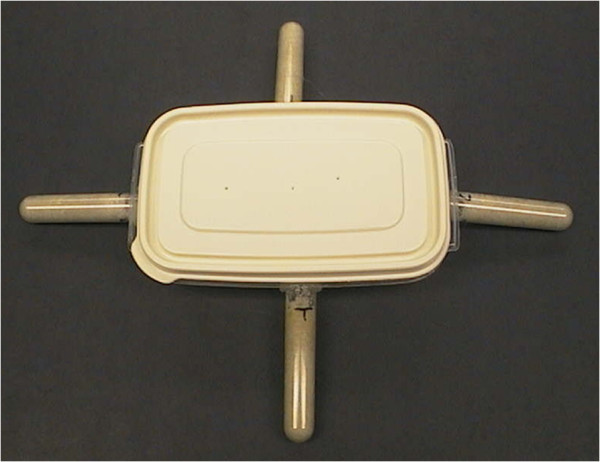
**Bioassay unit composed of a plastic container (14.5 × 8.5 × 4 cm) filled with 100 g of moistened sand, connected to four 14 ml polystyrene round bottom test tubes (17 × 100 mm) containing either treated (two tubes) or control (two tubes) substrate.**

Treated and control substrates were thoroughly moistened. A soil moisture meter (Spectrum Technologies, Plainfield, IL, USA) was used to establish moisture levels of 80% saturation for each substrate. Moist treated or control material was placed in 14 ml (17 × 100 mm) polystyrene round-bottom Falcon test tubes (Becton Dickinson, Franklin Lakes, NJ, USA). Each tube was filled with 10 ml of material. Tests were conducted using Rubbermaid™ storage containers (14.5 cm × 8.5 cm × 4 cm, Consolidated Plastics, Twinsburg, OH, USA) (Figure 4) [22]. Each container contained 100 g of sand (Standard Sand and Silica Company, Davenport, FL, USA) moistened with 20 ml of water. Each container had a 2 cm diameter hole on each side. A test tube was inserted into each hole and sealed in place using hot glue from a glue gun. For each container, there were two treatment tubes, which contained substrate treated with the stated microbe, and two control tubes, which contained substrate only. Because termites tend to aggregate, this experimental design reduced the probability that all of the termites would randomly aggregate in a single tube. Aggregation would impact the ability to attribute termite behavior to repellency [22]. The position of treatment and control tubes was alternated between replicates to preclude any positional effects. For each replicate, 200 termites (190 workers: 10 soldiers) were placed in the center of the container. Termites were able to move freely between the container and the tubes. For each experiment there were 12 replicates; four different colonies, with three replicates of each colony. Containers were kept in a dark environmental chamber at 28°C, 97% RH for 24 h. After 24 h, rubber stoppers were placed over the opening of each tube to prevent termites from leaving the tube while being counted. Each tube was removed from the container and all of the termites in each tube were counted. Numbers of termites in treated or control tubes for each replicate were determined.

For mortality bioassays, data were analyzed using analysis of variance (ANOVA) and least significant difference (LSD) at P≤ 0.05 [23]. All analyses were run using SAS Software. For repellency bioassays, differences in the number of termites in treated or control tubes were compared using a paired choice t-test.

Mention of trade names or commercial products in this article is solely for the purpose of providing specific information and does not imply recommendation or endorsement by the U.S. Department of Agriculture.

## Competing interests

The authors are employed by the organization that funded the project. The authors do not hold stock or shares in an organization that may benefit financially from the publication of this manuscript. No patents relating to this work are being applied for. The authors have no non-financial competing interests.

## Authors' contributions

MW carried out all microbial strain maintenance and propagation, mortality bioassays, and preparation of treated substrates. MC carried out all termite collection and maintenance, and repellency bioassays. MW and MC both analyzed statistics for their respective data.

## References

[B1] Culliney TW Grace JK Prospects for the biological control of subterranean termites (Isoptera: Rhinotermitidae), with special reference to Coptotermes formosanus Bull Entomol Res 2000 90 9 21 10.1017/S0007485300000663 10948359

[B2] Grace JK Approaches to biological control of termites Sociobiol 2003 41 115 121

[B3] Milner R Application of biological control agents in mound building termites (Isoptera: Termitidae) - Experiences with Metarhizium in Australia Sociobiol 2003 41 419 428

[B4] Myles TG Alarm, aggregation, and defense by Reticulitermes flavipes in response to a naturally occurring isolate of Metarhizium anisopliae Sociobiol 2002 40 243 255

[B5] Sun JZ Fuxa JR Richter A Ring D Interactions of Metarhizium anisopliae and tree-based mulches in repellence and mycoses against Coptotermes formosanus (Isoptera: Rhinotermitidae) Env Entomol 2008 37 755 763 10.1603/0046-225X(2008)37[755:IOMAAT]2.0.CO;2 18559182

[B6] Maketon M Sawangwan P Sawatwarakul W Laboratory study on the efficacy of Metarhizium anisopliae (Deuteromycota: Hyphomycetes) in controlling Coptotermes gestroi (Isoptera: Rhinotermitidae) Entomol Gen 2007 30 203 218

[B7] Wright MS Raina AK Lax AR A strain of Metarhizium anisopliae for controlling subterranean termites J Econ Entomol 2005 98 1451 1458 10.1603/0022-0493-98.5.1451 16334310

[B8] Wright MS Connick WJ Jackson MA Use of Paecilomyces spp. as pathogenic agents against subterranean termites 2003 1 17 US Patent No. 6660291

[B9] Dunlap CA Jackson MA Wright MA A foam formulation of Paecilomyces fumosoroseus, an entomopathogenic biocontrol agent Biocontrol Sci Technol 2007 17 513 523 10.1080/09583150701311614

[B10] Dunlap CA Jackson MA Wright MA Compositions of keratin hydrolysate and microbes for pest control applications 2012 1 12 US Patent No. 8263526

[B11] Luangsa-Ard JJ Hywel-Jones NL Manoch L Samson RA On the relationships of Paecilomyces sect. Isarioidea species Mycol Res 2005 109 581 589 10.1017/S0953756205002741 16018312

[B12] de Castilhos-Fortes R Matsumura ATS Diehl E Fiuza LM Susceptibility of Nasutitermes ehrhardti (Isoptera: Termitidae) to Bacillus thuringiensis subspecies Braz J Microbiol 2002 33 219 222 10.1590/S1517-83822002000300006

[B13] Mathew GM Lin SJ Chang JJ Huang CC DGGE detection and screening of lignocellulolytic bacteria from the termite gut of Coptotermes formosanus Malays J Microbiol 2011 7 201 209

[B14] Yanagawa A Yokohari F Shimizu S The role of antennae in removing entomopathogenic fungi from cuticle of the termite, Coptotermes formosanus J Insect Sci 2009 6 1 9 10.1673/031.009.0601 PMC3011873 19611249

[B15] Yanagawa A Shimizu S Resistance of the termite, Coptotermes formosanus Shiraki to Metarhizium anisopliae due to grooming BioControl 2007 52 75 85 10.1007/s10526-006-9020-x

[B16] Yanagawa A Yokohari F Shimizu S Defense mechanism of the termite, Coptotermes formosanus Shiraki, to entomopathogenic fungi J Invertebr Pathol 2008 97 165 170 10.1016/j.jip.2007.09.005 17949740

[B17] Su NY Scheffrahn RH A review of subterranean termite control practices and prospects for integrated pest management programmes Integr Pest Manag Rev 1998 3 1 13 10.1023/A:1009684821954

[B18] Wright MS Connick WJ Jr Jackson MA Use of Paecilomyces spp. as pathogenic agents against subterranean termites 2008 1 15 U.S. Patent No. 7390480

[B19] Jackson MA Mcguire MR Lacey LA Wraight SP Liquid culture production of desiccation tolerant blastospores of the bioinsecticidal fungus Paecilomyces fumosoroseus Mycol Res 1997 101 35 41 10.1017/S0953756296002067

[B20] Staples JA Milner RJ A laboratory evaluation of the repellency of Metarhizium anisopliae conidia to Coptotermes lacteus (Isoptera: Rhinotermitidae) Sociobiol 2000 36 133 148

[B21] Su NY Scheffrahn RH A method to access, trap, and monitor field populations of the Formosan subterranean termite (Isoptera: Rhinotermitidae) in the urban environment Sociobiol 1986 12 299 304

[B22] Cornelius ML Daigle DJ Connick WJ Parker A Wunch K Responses of Coptotermes formosanus and Reticulitermes flavipes (Isoptera: Rhinotermitidae) to three types of wood rot fungi cultures on different substrates J Econ Entomol 2002 95 121 128 10.1603/0022-0493-95.1.121 11942746

[B23] Cody RP Smith JK Applied Statistics and the SAS Programming Language 1997 NJ: Prentice-Hall Inc

